# CPP alters theta/gamma oscillations in rat hippocampus: simulation and experiment

**DOI:** 10.1186/1471-2202-13-S1-P91

**Published:** 2012-07-16

**Authors:** Mohamed A Sherif, Jeremy M Barry, Samuel A Neymotin, William W Lytton

**Affiliations:** 1 Department of Psychiatry, SUNY Downstate Medical Center, Brooklyn, NY 11203, USA; 2 Neurosimulation Lab, SUNY Downstate Medical Center, Brooklyn, NY 11203, USA; 3 Kings County Hospital, Brooklyn, NY 11203, USA; 4 Epilepsy, Cognition and Development group at Darmouth Hitchcock Medical Center, Lebanon, NH 03766, USA

## 

Several different NMDA receptor (NMDAR) antagonists are used to produce pharmacological models for schizophrenia. These antagonists have different molecular mechanisms of action: non-competitive (e.g. ketamine, which binds at a site different from glutamate) and competitive (e.g. CPP, which displaces glutamate). Because competitive antagonism results in more glutamate availability, this may result in glutamate spill-over either onto other receptors of the same synapse or to neighboring synapses. In this study, we modeled the effects of competitive blockade by simultaneously blocking NMDAR and increasing activity of AMPAR (spillover). We looked at alterations of network oscillations and synchronization using our biophysical model of the CA1 region of hippocampus using the NEURON simulator. We then compared the results with data obtained from the CA1 region of hippocampus in rats injected with CPP.

Our network consisted of 800 five-compartment pyramidal cells (PYR), 200 one-compartment basket cell interneurons (BAS), and 200 one-compartment oriens lacunosum-moleculare interneurons (OLM) [1]. All cells contained leak current, transient sodium current and delayed rectifier current. Additionally, pyramidal cells contained potassium type A current and pyramidal and OLM cells had Ih current. Cell classes were interconnected probabilistically with AMPA/NMDA synapses, and two classes of GABAa synapses. The OLM cells formed synapses on the distal dendrites of pyramidal cells, while the basket cells synapsed proximally on pyramidal and other basket cells. Pyramidal cells synapsed on both types of interneurons with AMPA/NMDA synapses. All synapses were bombarded with external Poisson inputs to generate network activity. Competitive inhibition was modeled by setting the conductance across NMDAR to zero, and increasing current conductance across AMPAR on the same set of synapses. We modeled this on synaptic sites on all cell populations. Also, given the possible variation of the affinity of competitive inhibitors to NMDAR on different cells, we studied this effect when it took place at the receptors of the 3 different cell populations separately. Experimental recordings were made from tetrode arrays implanted in the CA1 region of Long Evans rats chasing sugar pellets in a box. CPP at a dose of 5 mg/kg was injected intraperitoneally in the rats. Recordings were for 16 min sessions, separated by 30 min breaks during which the rats were returned to their cages.

Recordings from CA1 of rat hippocampus under the effect of CPP, showed that there is a reduction in theta and an increase in gamma. Modeling competitive inhibition to glutamate at all synapse locations (on OLM, BAS, and PYR) resulted in a reduction in the theta power and an increase in gamma power, differing from the noncompetitive effect with ketamine, which reduced both theta and gamma when given across all synapses. However, this result was also seen when the effect of competitive inhibition was restricted to only OLM cell NMDARs. Therefore, our model demonstrates both of these interpretations of CPP action. We will examine further correlates between simulation and experiment to reduce this uncertainty.
